# Effects of deficiency in the *RLBP1*-encoded visual cycle protein CRALBP on visual dysfunction in humans and mice

**DOI:** 10.1074/jbc.RA120.012695

**Published:** 2020-03-18

**Authors:** Jose Ronaldo Lima de Carvalho, Hye Jin Kim, Keiko Ueda, Jin Zhao, Aaron P. Owji, Tingting Yang, Stephen H. Tsang, Janet R. Sparrow

**Affiliations:** ‡ Department of Ophthalmology, Columbia University Irving Medical Center, New York, New York 10032; § Department of Pharmacology, Columbia University Irving Medical Center, New York, New York 10032; ¶ Department of Pathology and Cell Biology, Columbia University Irving Medical Center, New York, New York 10032

**Keywords:** retina, retinal degeneration, retinoid, vitamin A, vision, bisretinoids, CRALBP, retinitis punctata albescens, RLBP1, visual cycle

## Abstract

Mutations in *retinaldehyde-binding protein 1* (*RLBP1*), encoding the visual cycle protein cellular retinaldehyde-binding protein (CRALBP), cause an autosomal recessive form of retinal degeneration. By binding to 11-*cis*-retinoid, CRALBP augments the isomerase activity of retinoid isomerohydrolase RPE65 (RPE65) and facilitates 11-*cis*-retinol oxidation to 11-*cis*-retinal. CRALBP also maintains the 11-*cis* configuration and protects against unwanted retinaldehyde activity. Studying a sibling pair that is compound heterozygous for mutations in *RLBP1*/CRALBP, here we expand the phenotype of affected individuals, elucidate a previously unreported phenotype in *RLBP1*/CRALBP carriers, and demonstrate consistencies between the affected individuals and *Rlbp1*/Cralbp^−/−^ mice. In the *RLBP1*/CRALBP-affected individuals, nonrecordable rod-specific electroretinogram traces were recovered after prolonged dark adaptation. In ultrawide-field fundus images, we observed radially arranged puncta typical of *RLBP1*/CRALBP-associated disease. Spectral domain-optical coherence tomography (SD-OCT) revealed hyperreflective aberrations within photoreceptor-associated bands. In short-wavelength fundus autofluorescence (SW-AF) images, speckled hyperautofluorescence and mottling indicated macular involvement. In both the affected individuals and their asymptomatic carrier parents, reduced SW-AF intensities, measured as quantitative fundus autofluorescence (qAF), indicated chronic impairment in 11-*cis*-retinal availability and provided information on mutation severity. Hypertransmission of the SD-OCT signal into the choroid together with decreased near-infrared autofluorescence (NIR-AF) provided evidence for retinal pigment epithelial cell (RPE) involvement. In *Rlbp1*/Cralbp^−/−^ mice, reduced 11-*cis*-retinal levels, qAF and NIR-AF intensities, and photoreceptor loss were consistent with the clinical presentation of the affected siblings. These findings indicate that *RLBP1* mutations are associated with progressive disease involving RPE atrophy and photoreceptor cell degeneration. In asymptomatic carriers, qAF disclosed previously undetected visual cycle deficiency.

## Introduction

Vision is initiated by the absorption of a photon of light by the opsin chromophore 11-*cis*-retinaldehyde in the outer segments of rod and cone photoreceptor cells. This photon capture causes isomerization of 11-*cis*-retinaldehyde to the bleached product all-*trans*-retinaldehyde. For continued light detection, a supply of 11-*cis*-retinal is provided to rod and cone photoreceptor cells by a multistep enzyme pathway, the visual cycle (retinoid cycle), that reconverts all-*trans*-retinal to light-sensitive 11-*cis*-retinal. Cellular retinaldehyde-binding protein (CRALBP),^3^ a 36-kDa aqueous soluble carrier, participates in this process (1). CRALBP is expressed in abundance by both retinal pigment epithelial (RPE) and Muller cells of retina and is important for rod- and cone-driven vision (2).

In humans, mutations in *retinaldehyde-binding protein 1* (*RLBP1*), the gene encoding CRALBP, cause autosomal recessive retinal diseases with phenotypes described as retinitis punctata albescens (RPA), autosomal recessive retinitis pigmentosa (arRP), Bothnia dystrophy, and Newfoundland rod-cone dystrophy (2–9). The variable presentation likely reflects, at least in part, the effect of particular mutations on protein structure and function (8). The *RLBP1*/CRALBP phenotype is characterized by varying degrees of early onset delayed dark adaptation, abnormal electroretinographic (ERG) responses, loss of peripheral vision, and progressive macular atrophy (10, 11). Also a characteristic of CRALBP deficiency is the presence of white dot-like aberrations within the fundus (11, 12); these puncta are visible in color fundus and short-wavelength fundus autofluorescence (SW-AF) images (11, 12). RPE atrophy is accompanied by hypertransmission of the spectral domain optical coherence tomography (SD-OCT) signal into the choroid (10). It is estimated that *RLBP1*-associated disease accounts for 0.5% of all RP cases (13), a condition that has a prevalence of 1 in 4000 in developed countries (14).

CRALBP in RPE cells participates in supplying 11-*cis*-retinal to both rods and cones, whereas cones also rely on 11-*cis* chromophore present in Muller cells (15–17). CRALBP performs multiple functions in the visual cycle. First, by noncovalently binding 11-*cis*-retinol as it is produced by the isomerase RPE65, CRALBP reduces product inhibition of the isomerase activity (18, 19) thereby ensuring efficient production of 11-*cis*-retinol. CRALBP can bind both 11-*cis*-retinol and 11-*cis*-retinal (16) thereby facilitating the oxidation of protein-bound 11-*cis*-retinol to 11-*cis*-retinal, catalyzed by retinol dehydrogenase 5 (RDH5) (16, 20). Accordingly, delayed dark adaptation is exhibited by human patients carrying a mutation of the *RLBP1*/CRALBP gene and by Cralbp^−/−^ mice (1, 2, 9). Additionally, the hydrophobic pocket of CRALBP imposes a conformational strain on 11-*cis*-retinal and thus maintains the isomeric state of 11-*cis*-retinal even in the presence of light (21–24). As a retinaldehyde carrier CRALBP also protects against unwanted aldehyde toxicity (16).

Here we report findings in two siblings with early-onset retinal disorders who were compound heterozygous for two pathogenic mutations in the *RLBP1* gene. Previously unreported features of *RLBP1*/CRALBP disease in the affected patients are presented and replication in Cralbp^−/−^ mice is demonstrated. Importantly, to the best of our knowledge, this is the first work to show a phenotype in asymptomatic human carriers of *RLBP1*/CRALBP mutations.

## Results

### RLBP1/CRALBP-associated disease in affected human patients

**Figure 1.**
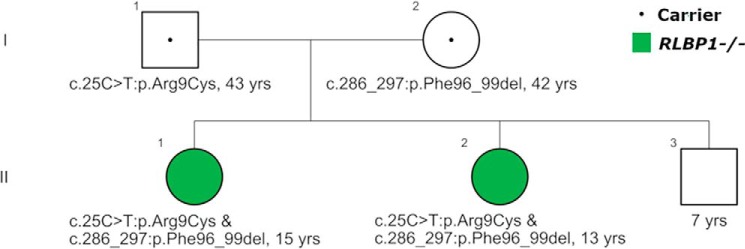
**Autosomal recessive inheritance patterns.** Affected female siblings (II-1; II-2; *green filled circles*) compound heterozygous for the missense multation c.25C>T:pArg9Cys and c.286_297:p.Phe96_99 deletion in the *RLBP1* gene. Two generations (*I*, *II*) are shown. Male, *square*; female, *circl*e. Retinitis punctata albescens patients, *green filled symbol*; carriers, *dot in symbol*; unknown, *unfilled symbol*.

A family with two clinically affected female siblings (Fig. 1), a 15-year-old (II-1) and a 13-year-old (II-2), presented to the Department of Ophthalmology at Columbia University Irving Medical Center with a complaint of photophobia and poor night vision over the prior year. The younger brother (II-3) and the parents (I-1 and I-2) were asymptomatic. Whole exome sequencing of DNA obtained from peripheral blood revealed that both children were compound heterozygous for the missense mutation c.25C>T:p.Arg9Cys and c.286_297:p.Phe96_99 deletion in the *RLBP1* gene and diagnosis of RPA was made. These mutations have been reported previously (11, 25). Concurrent genetic analyses of the parents revealed that the father is heterozygous for the c.25C>T:p.Arg9Cys mutation and the mother is heterozygous for the c.286_297:p.Phe96_99del variant (Fig. 1). Clinical and genetic characteristics of the patients and parents are summarized in Table 1.

**Table 1:** **Clinical and genetic characteristics of the family members**

Patient	Gender	Age	Clinical symptoms	BCVA*^a^* (logMAR)		qAF8		Genetics
				OD	OS	OD	OS	
I-1	M	43	No symptoms	0.0	0.0	183	155	*RLBP1*^+/−^ c.25C>T:p.Arg9Cys
I-2	F	42	No symptoms	0.0	0.0	114	118	*RLBP1*^+/−^ c.286_297:p.Phe96_99del
II-1	F	15	Photophobia, poor night vision	0.06	0.06	–	–	*RLBP1*^−/−^ c.25C>T:p.Arg9Cys and c.286_297:p.Phe96_99del
II-2	F	13	Photophobia, poor night vision	0.06	0.24	–	–	*RLBP1*^−/−^ c.25C>T:p.Arg9Cys and c.286_297:p.Phe96_99del
II-3	M	7	No symptoms	0.02	0.02	N/A	N/A	NA*^b^*

*^a^* BCVA, best corrected visual acuity; logMAR, logarithm of the minimum angle of resolution; OD, right eye; OS, left eye; qAF8, average quantitative autofluorescence of the 8 segments of the ring at an eccentricity of approximately 7° to 9° from the fovea.

*^b^* NA, not applicable.

### Full-field electroretinogram (ffERG)

**Figure 2.**
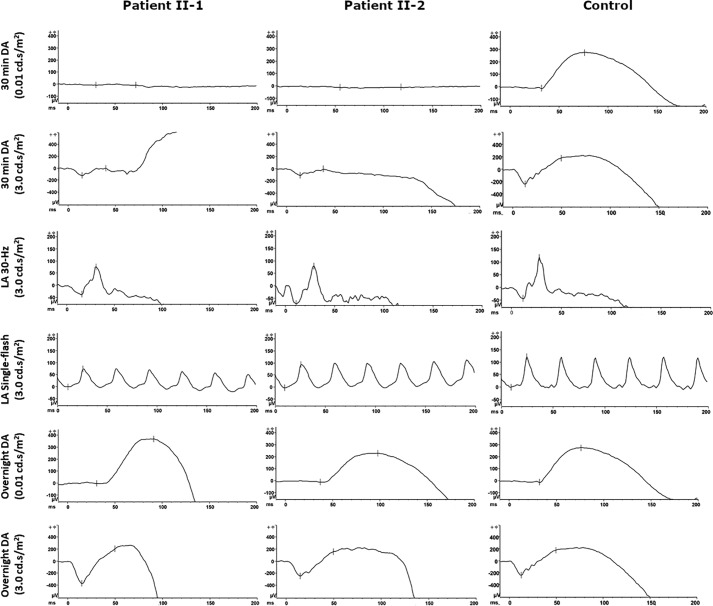
**Rod-cone dysfunction in ffERG.** Patients II-1 and II-2: rod-specific ERG with 0.01 cd.s/m^2^ stimulus following standard 30-min dark adaptation was absent. Maximum ERG (3.0 cd.s/m^2^) had a diminished response. Photopic ERG was within the normal range. After prolonged dark-adaptation (>8 h overnight eye-patch), rod-specific ERG and maximum ERG responses were recovered. Normal control is shown in the *right column. DA*, dark-adapted; *LA*, light-adapted.

ffERGs were performed in both scotopic and photopic states. Standard dark-adapted (30 min) rod-specific ERGs were extinguished in both daughters (II-1 and II-2; Fig. 2, *top rows*). Light-adapted 30-Hz flicker and single-flash were within the normal range (Fig. 2, *middle rows*). Interestingly, after prolonged dark-adaptation (>8 h overnight eye-patch), the rod responses measured by ERG increased to the normal range (Fig. 2, *bottom rows*). The young brother (II-3) had normal ERG results (not shown).

### Fundus imaging

**Figure 3.**
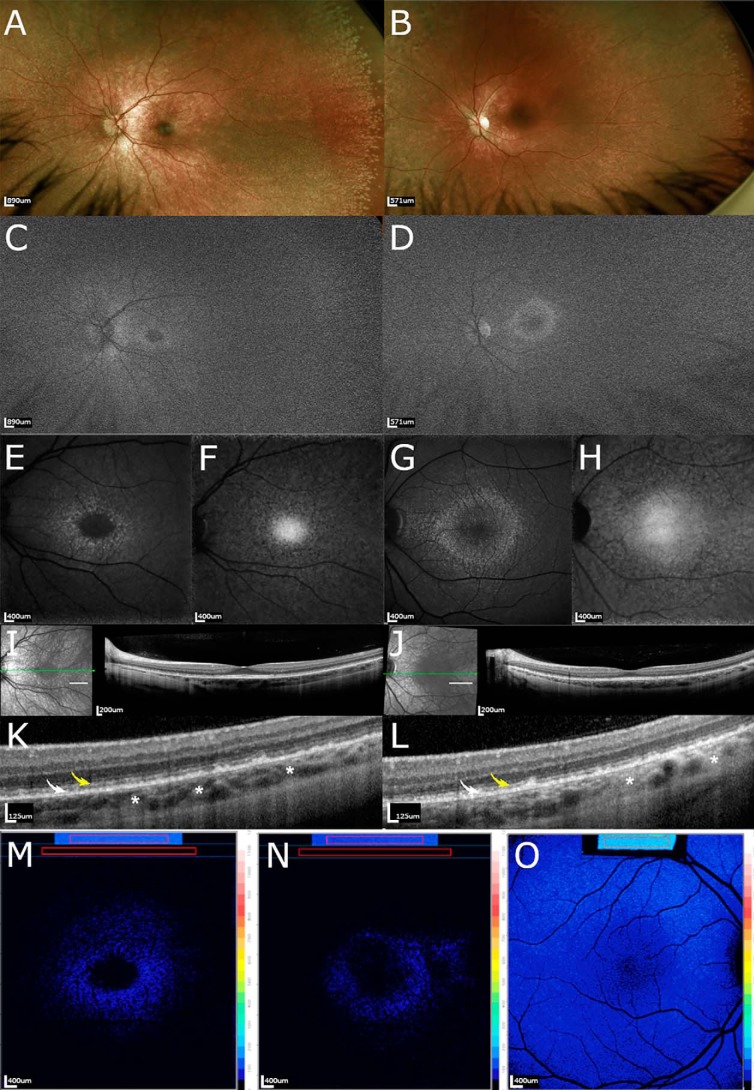
**Multimodal images in siblings deficient in CRALBP.** Images of II-1 (*left*) and II-2 (*right*) both of whom are compound heterozygous for mutations in the *RLBP1* gene. *A* and *B,* UWF color fundus photographs. Note the radially arranged *white dots* more visible in the peripheral retina. *C* and *D,* green UWF autofluorescence. Fovea was detected as a small dark zone with surrounding speckled brightness. *E* and *G,* SW-AF. A ring of speckled hyperautofluorescence transitioned into faintly autofluorescent macular spots and mottling at greater eccentricity. *F* and *H,* NIR-AF. The fovea exhibits the characteristic elevated NIR-AF intensity. Hypoautofluorescent dots surrounded by areas of normal fluorescence in the parafoveal region can be appreciated. *I*, *J*, and *K*, *L,* SD-OCT. Note undulations of the ellipsoid zone (*yellow arrows*), hyperreflective irregularities in the interdigitation zone (*white arrows*), and foci of hypertransmission into the choroid (*white asterisks*). *K* and *L* represent magnified images of the area indicated by the *short white lines* in *I* and *J. M–O*, color coded quantitative fundus autofluorescence images. Patients II-1 (*M*) and II-2 (*N*) had undetectable levels of AF except for a parafoveal ring. A healthy control eye 11-year-old subject is shown (*O*).

In ultrawide-field (UWF) pseudo-color fundus images (200°), the patients exhibited radially arranged white spots that were more visible in peripheral retina (Fig. 3, *A* and *B*). The white spots were also more pronounced in the older sibling. UWF-autofluorescence/green (UWF-AF/green) images using 532 nm excitation provided images that were largely grainy but the optic disk and retinal vessels were visible (Fig. 3, *C* and *D*). The fovea was detected as a small dark zone with surrounding speckled brightness. The horizontally oriented oval-shaped central zone of increased brightness that in healthy eyes envelopes the macula and optic disk and is typical of UWF-AF/green fundus images (26) was less pronounced.

In both siblings, SW-AF (blue) images acquired with 488 nm excitation presented with typical central hypoautofluorescence but contrasting foveal darkness was not evident (Fig. 3, *E* and *G*). Outside the zone of reduced autofluorescence was a ring of speckled autofluorescence that at greater eccentricity transitioned into faintly autofluorescent macular spots and mottling.

Near-infrared autofluorescence (NIR-AF) fundus images obtained from both children presented with the central elevated intensity that is characteristic of healthy eyes and that is attributable to increased melanin optical density (Fig. 3, *F* and *H*). Hypoautofluorescent dots surrounded by areas of apparently normal fluorescence were present in the parafoveal region.

In SD-OCT scans, hyperreflective aberrations were observed in II-1 and II-2 (Fig. 3, *I* and *K*, *J* and *L*). These changes were characterized by undulations and discontinuities of the ellipsoid zone (*yellow arrows*), hyperreflective irregularities in the interdigitation zone (*white arrows*), and foci of hypertransmission into the choroid (*white asterisks*). These abnormalities were observed in both siblings and were more pronounced in nonfoveal macula (Fig. 3, *K* and *L*). In both patients the external limiting membrane was continuous.

### Quantitation of fundus images in affected patients and carrier parents

SW-AF originates from bisretinoid by-products of the visual cycle. To assess SW-AF intensities, the quantitative fundus autofluorescence (qAF) approach was used to measure intensities in the two patients and their parents. In the two *RLBP1*/CRALBP-affected siblings, qAF intensities within the concentric segments positioned at an eccentricity of 7–9° were not measurable. This profound and uniform reduction in qAF was evident in qAF color-coded images (Fig. 3, *M* and *N*) when comparison was made to a healthy control eye of similar age (Fig. 3*O*).

**Figure 4.**
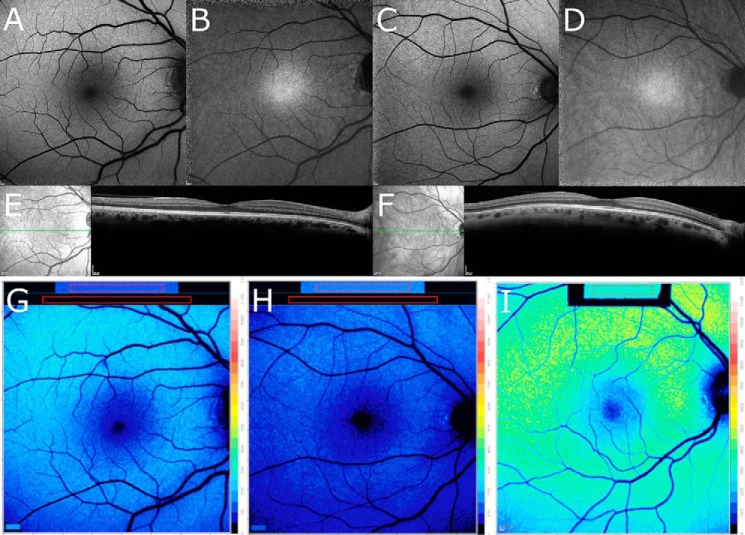
**Fundus images of the parents who are carriers of mutations in *RLBP1/*CRALBP.** Subjects I-1 (*left*) and I-2 (*right*). Subject I-1 is heterozygous for c.25C>T mutation in the *RLBP1* gene and subject I-2 is heterozygous for the c.286_297del variant in the *RLBP1* gene. The C.289_297:p.Phe96_99 deletion carrier has less autofluorescence than the missense mutation c.25C>T:p.Arg9Cys carrier. *A* and *C,* SW-AF. Images acquired in normalized mode do not reveal differences in gray level intensities. *B* and *D,* NIR-AF. The fovea exhibits typical elevated intensity; contrast with AF in periphery appears to be increased. *E* and *F,* SD-OCT. Reflectivity layers appear normal. *G–I*, color-coded qAF images of the carriers I-1 (*G*), I-2 (*H*), and an age-matched healthy eye (*I*). Reduced qAF characterizes the symptomless carriers.

**Figure 5.**
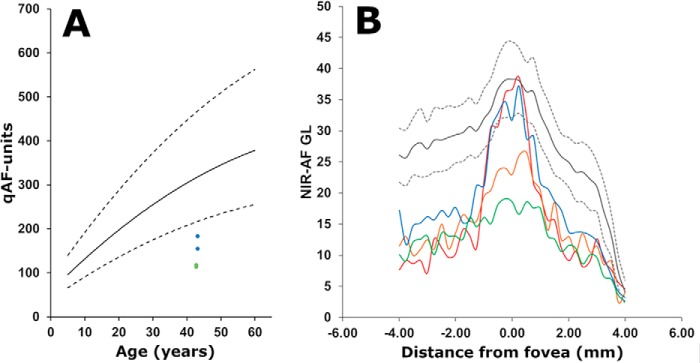
**Quantitation of fundus autofluorescence.** *A,* short-wavelength (488 nm) fundus autofluorescence measured as qAF at an eccentricity of 7–9° and plotted as a function of age. Mean qAF_8_ of parents (*blue*, I-1; and *green* I-2) are plotted together with mean (*solid black line*) and 95% confidence levels (*dashed lines*) acquired from eyes of healthy subjects. Note that qAF intensities at the same location in the affected siblings were not measurable. *B,* semi-quantitative NIR-AF intensity profiles plotted as a function of temporal-to-nasal (left to right) distance (mm) along a horizontal line through the fovea (0). *Gray lines* represent mean (*solid lines*) and 95% confidence intervals (*dashed lines*) of healthy control eyes. Subjects are represented as follows: *blue line*, I-1; *green line*, I-2; *red line*, II-1; *orange line*, II-2.

Importantly, although the carrier parents did not present with visual symptoms and SW-AF, NIR-AF and SD-OCT images were unremarkable (Fig. 4, *A–F*), in both parents color-coded qAF images revealed lower SW-AF intensities (Fig. 4, *G* and *H*) than in an age-similar healthy eye (Fig. 4*I*). The qAF value calculated as the mean of the 8 concentric segments was below the 95% confidence interval of healthy eyes (Fig. 5*A*). qAF was also lower in the mother who carried the c.286_297:p.Phe96_99 deletion than in the father who was a heterozygous carrier of the c.25C>T:p.Arg9Cys mutation.

Melanin-derived NIR-AF (27–30) intensities originating primarily in RPE were measured along a horizontal axis through the fovea and were compared with the measurements obtained from a group of 19 healthy subjects that served as control. Interestingly, intensities in the NIR-AF profiles were decreased in both the probands and heterozygous parents (Fig. 5*B*); this reduction is indicative of RPE involvement in the disease processes. It is important to note that as with qAF, the reduction in the central NIR-AF signal was more pronounced in the carrier (mother) bearing the c.286_297:p.Phe96_99 deletion than in the carrier (father) harboring the missense mutation c.25C>T:p.Arg9Cys carrier. The affected siblings were compound heterozygous and exhibited a difference in the NIR-AF profiles. Although younger, the 13-year-old sibling presented with a greater decrease in NIR-AF. This finding deserves further investigation.

### Cralbp^−/−^ mice: UPLC measurement of retinoids

**Figure 6.**
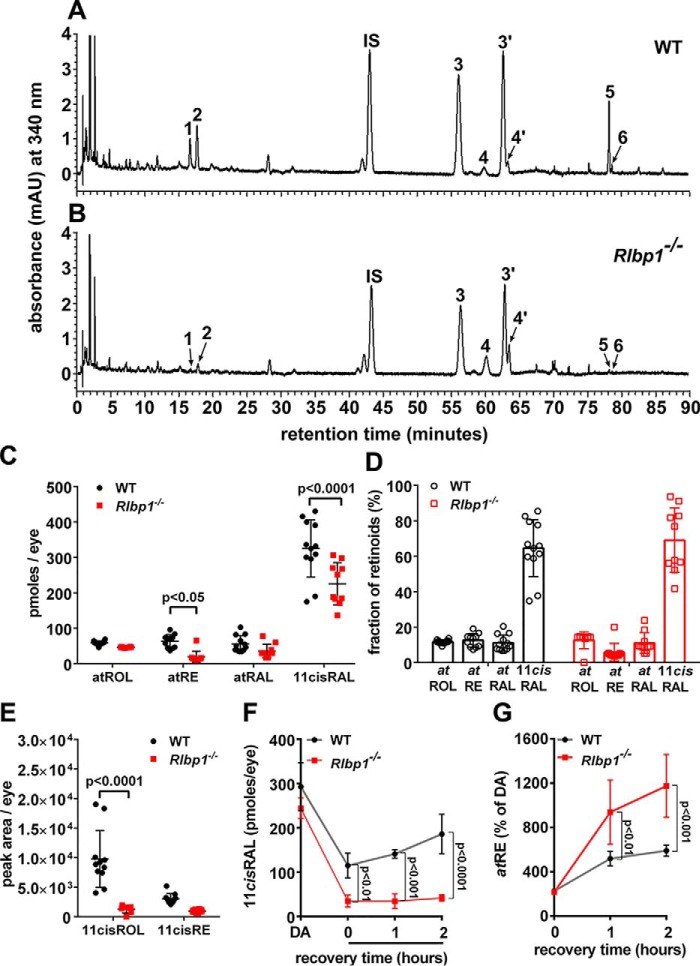
**Retinoid levels in dark-adapted agouti Cralbp^−/−^ and WT mice.** Ages were 2–4 months. *A* and *B*, representative UPLC chromatograms demonstrating separation of hexane-extracted retinoids in eyes of agouti WT (*A*) and Cralbp^−/−^ mice (*B*). Monitoring was done at 340 nm. Age 4 months; 1 eye/sample. The *numbers* indicate the identity of peaks based on comparison with standards. *1*, 11-*cis*-retinol; *2*, all-*trans-*retinol; *IS,* internal standard, all-*trans*-retinyl acetate; *3*, *anti* 11-*cis*-retinal-(*O*-ethyl) oxime; *4*, *anti* all-*trans*-retinal-(*O*-ethyl) oxime; 3′, *syn* 11-*cis*-retinal-(*O*-ethyl) oxime; 4′, *syn* all-*trans*-retinal-(*O*-ethyl) oxime; *5*, all-*trans*-retinyl palmitate; *6*, 11-*cis*-retinyl palmitate. *C* and *D,* quantitation of retinoids as picomoles/eye (*C*). Presentation of data in *C* as percentage of total retinoid (*D*). All-*trans*-retinol (*atROL*), all-*trans*-retinyl palmitate (*atRE*), all-*trans*-retinal (*atRAL*), 11-*cis*-retinal (*11cisRAL*) are shown. *E,* quantitation of 11-*cis*-retinol (*11cisROL*) and 11-*cis*-retinyl palmitate (*11cisRE*) as peak area/eye. *F* and *G,* levels of 11-*cis*-retinal (*F*) and all-*trans*-retinyl ester (*G*) in dark-adapted (*DA*) mice recovering from a photobleach for 1 and 2 h in the dark. Mean ± S.D., 10–12 samples (*C–E*); 4 samples (*F* and *G*); *p* values determined by ANOVA and Sidak's multiple comparisons test.

CRALBP deficiency does not block the functioning of the visual cycle but is considered to modulate its kinetics (2). Thus to test the efficiency of CRALBP activity, we measured retinoid levels in dark-adapted mice (Fig. 6, *A* and *B*). To enable extraction efficiency the nucleophile hydroxylamine was used to promote 11-*cis*-retinal release from the stable retinal oxime derivatives (anti and syn) (λ_max_, 367 nm) (31). Measurements of 11-*cis*-retinal in 2-month-old dark-adapted WT mice were similar to values previously reported (32, 33). Meanwhile in Cralbp^−/−^ mice, 11-*cis*-retinal measured as picomoles per eye was significantly lower than in WT mice (*p* < 0.001, ANOVA and Sidak's multiple comparison test were used) (Fig. 6*C*). However, expressed as a fraction of total retinoid there was no significant difference (Fig. 6*D*), as was previously reported (1). Amounts of 11-*cis*-retinol were also lower in the Cralbp^−/−^ mice (Fig. 6*E*).

We also monitored the recovery of retinoid levels following visual pigment bleaching (white light, 8200–900 lux, 3 min) in dark-adapted *Rlbp1*/Cralbp^−/−^ and WT mice. With 1 and 2 h in the dark after photobleaching, there was no evidence of recovery of 11-*cis*-retinal levels in the *Rlbp1*/Cralbp^−/−^ mice and 11-*cis*-retinal was significantly lower than in WT mice (Fig. 6*F*). Conversely, all-*trans*-retinyl ester amounts were significantly higher at 1 and 2 h after the photobleach (Fig. 6*G*). As a percent of dark-adapted pre-bleach levels, all-*trans*-retinal was higher immediately after photobleaching (data not shown).

### Fundus imaging in mice

**Figure 7.**
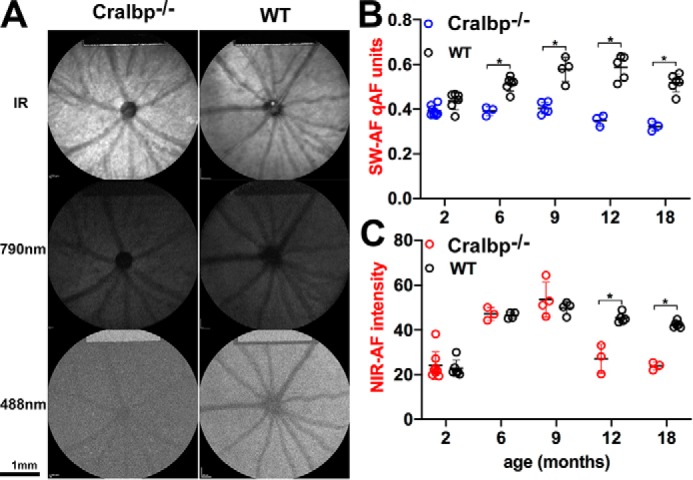
**Fundus images acquired from agouti Cralbp^−/−^ and WT mice.** *A,* IR reflectance (IR; 820 nm), SW-AF (488 nm), and NIR-AF (790 nm) images acquired from agouti Cralbp^−/−^ and WT mice (age 6 months). *B,* SW-AF intensities were measured in agouti Cralbp^−/−^ and WT mice with qAF protocols and plotted as a function of age. Gray levels were measured 8.25–19.25° from the disc center and were normalized to gray levels in an internal fluorescent reference (rectangular area at top of image) to calculate qAF. Means of 3–8 mice; *, *p* < 0.05, ANOVA and Sidak's multiple comparisons test. *C,* NIR-AF intensities were measured in agouti Cralbp^−/−^ and WT mice. Mean ± S.D. of 3–8 mice; *, *p* < 0.05, ANOVA and Sidak's multiple comparisons test.

In the Cralbp^−/−^ and WT mice, we imaged *in vivo* fundus autofluorescence using instrumentation similar to that employed clinically. SW-AF (488 nm) images were uniformly low in intensity and due to poor contrast blood vessels were muted (Fig. 7*A*). The brightness of the internal fluorescent reference in the qAF images (488 nm) attested to the long exposure time used to generate the fundus AF in these mice. Puncta were not observed in the fundus.

Measurement of SW-AF by qAF revealed that fundus intensities were significantly lower in the Cralbp^−/−^ mice as compared with the agouti 129S1/SvImJ mice from age 6 to 18 months (Fig. 7*B*). qAF in the Cralbp^−/−^ mice also declined at 9 months of age and was significantly lower at 18 months (*p* < 0.05).

NIR-AF intensities in Cralbp^−/−^ mice at 6 and 9 months of age were similar to that of agouti WT at ages 2, 6, and 9 months (Fig. 7*C*). Levels then dropped significantly at 12 and 18 months of age. In Cralbp^−/−^ mice NIR-AF intensities at 12 and 18 months were significantly lower than in WT (Fig. 7*C*).

### High performance LC (HPLC)

**Figure 8.**
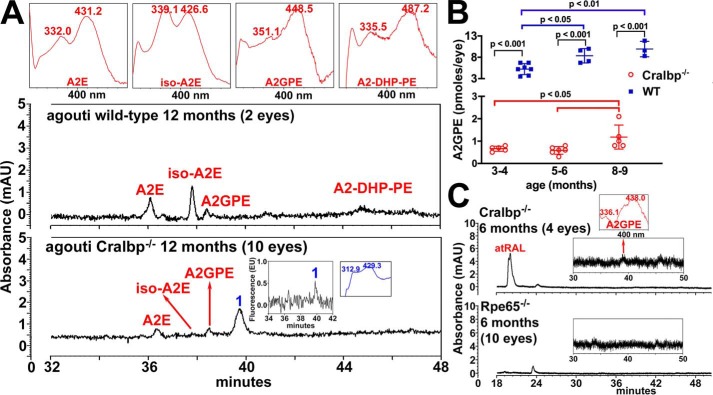
**Reduced bisretinoids in Cralbp^−/−^ mice retinae.** HPLC analysis of bisretinoids in WT and Cralbp^−/−^ mice. *A,* representative reverse phase HPLC chromatograms with monitoring at 430 nm, illustrate the detection of the bisretinoids identified on the basis of UV-visible absorbance and retention times (*Rt*) recorded using authentic standards. A2E (Rt 36.5 min), iso-A2E (Rt 37.8 min), A2GPE (Rt 38.4 min), A2-DHP-PE (Rt 45.1 min) and oxidized bisretinoid (1, Rt 39.7 min). Age was 12 months. *Insets*: *top,* UV-visible absorbance spectra of chromatographic peaks corresponding to bisretinoids, *inset*, fluorescence at λ_ex_ = 430 nm/λ_em_ = 600 nm and UV-visible absorbance of peak 1. *B,* quantification of bisretinoid in Cralbp^−/−^ and WT mice. *C,* A2GPE is not detectable in Rpe65^−/−^ mouse eyecups analyzed by UPLC. Eyecups from pigmented Cralbp^−/−^ and WT mice were analyzed by UPLC with a BEH phenyl column and monitoring at 430 nm. Values are mean ± S.D. of 3–7 independent samples. 2–4 eyes/sample. *p* values were determined by one-way ANOVA and Tukey's multiple comparison tests.

Because SW-AF was reduced in the Cralbp^−/−^ mice we also measured the bisretinoid lipofuscin fluorophores that form as a by-product of the visual cycle and that serve as the source of this SW-AF emission. A representative HPLC chromatogram in Fig. 8 illustrates the detection of bisretinoids in an agouti WT mouse (2 eyes/sample). With identification using UV-visible absorbance spectra (monitoring at 430 nm) and retention times determined from authentic compound, elution peaks were assigned to the bisretinoids A2E (λ_max_ = 431, 332), iso-A2E (λ_max_ = 426, 339), A2GPE (A2-glycerophosphoethanolamine) (λ_max_ = 448, 351), and A2-DHP-PE (A2-dihydropyridinephosphatidylethanolamin) (λ_max_ = 487, 335) (Fig. 8*A*). Conversely, even in an extract from 10 eyes pooled from Cralbp^−/−^ mice, A2E and iso-A2E were consistently less abundant than in a sample of 2 eyes from WT mice (Fig. 8*A*).

Because ultra-performance LC (UPLC) provides excellent sensitivity and resolution with smaller sample sizes than HPLC, we used UPLC for further quantitative analysis. A2GPE increased with age in both the Cralbp^−/−^ and WT mice (Fig. 8*B*), but at all ages A2GPE was also significantly lower in Cralbp^−/−^ than WT.

RPE65 is the isomerohydrolase responsible for converting all-*trans*-retinyl-ester to 11-*cis-*retinol within RPE cells (34–36) and *RLBP1*/CRALBP-associated disease is considered to share clinical features with *RPE65*-related dystrophy (37, 38). Rpe65 null mutant (Rpe65^−/−^) mice do not generate 11-*cis*-retinal chromophore (39). A comparison of 6-month-old Cralbp^−/−^ and Rpe65^−/−^ mice (Fig. 8*C*) revealed that whereas A2GPE was reduced in Cralbp^−/−^ mice, an A2GPE peak was undetectable in the Rpe65^−/−^ mice even in a sample of 10 eyes.

### Analysis of outer nuclear layer (ONL) in Cralbp^−/−^ mice

**Figure 9.**
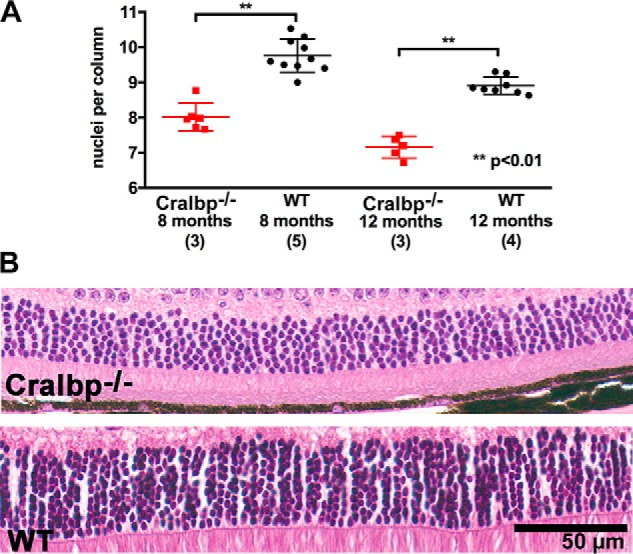
**Photoreceptor cell loss in Cralbp^−/−^ mice.** *A,* numbers of nuclei per column extending the width of the ONL. Mean ± S.D.; Cralbp^−/−^, 6 eyes (age 8–9 months), 5 eyes (age 12 months); WT: 10 eyes (age 8–9 months); 8 eyes (12 months). **, *p* < 0.01, ANOVA and Tukey's multiple comparisons test. *B,* representative light micrographs of inferior hemiretina of Cralbp^−/−^ and WT mice (age 12 months) within 500–1000 μm from the optic nerve head.

Because retinoids are also required to sustain the viability of photoreceptor cells (1, 16) we examined photoreceptor cell numbers by counting nuclei in the ONL (Fig. 9). The numbers of photoreceptor cell nuclei spanning the width of the outer nuclear layer in the WT mice at the age of 8–9 months was 10.0 (± 0.38, S.D.) (Fig. 9*A*); this value was typical of the 9–11 range reported in the literature (40–47). In the Cralbp^−/−^ mice at 8–9 months of age the mean number of nuclei was reduced to 8.6 (± 0.48, S.D.) and at age 12 months a further reduction to 7.32 (± 0.44, S.D.) (*p* < 0.05; two-way ANOVA and Sidak's multiple comparisons test) occurred (Fig. 9, *A* and *B*).

## Discussion

We have presented the clinical and molecular analyses of two siblings compound heterozygous for disease-causing mutations in the *RLBP1* gene, their carrier parents, and Cralbp^−/−^ mice. By employing multiple imaging modalities, we observed that the dot-like puncta characteristic of color fundus images in *RLBP1-*disease (11, 12) were prominent and radially arranged in peripheral retina when viewed by UWF imaging. These puncta were also autofluorescent in SW-AF images and hypofluorescent in NIR-AF images. The SD-OCT scans presented foci of abnormal outer retinal reflectivity together with hypertransmission of SD-OCT signal into the choroid. Full-field ERG recordings revealed that rod responses in the affected siblings were severely depressed. Nevertheless, to our knowledge this is the first report to show that after prolonged dark-adaptation the patients with RPA recovered their rod-specific ERG responses. This finding together with reduced levels of 11-*cis*-retinal and 11-*cis*-retinol in the Cralbp^−/−^ mice confirmed that a deficiency in CRALBP does not block the enzymatic production of 11-*cis*-retinoid but 11-*cis*-retinal is regenerated at a slower rate. The reduction in SW-AF intensity measured as qAF in the affected adolescents, the two carriers and Cralbp^−/−^ mice are corroborated by HPLC quantitation of bisretinoids in the mice and results from reduced availability of visual chromophore.

We gained insight into the structural correlates of the white spots. The hypopigmentation evinced as white dots in pseudocolor fundus images and as reduced melanin signal (27–30) in NIR-AF images indicated a change in the RPE monolayer, whereas hypertransmission of the SD-OCT signal posterior to the RPE was indicative of RPE atrophy at these positions. We have previously observed that outside the macula of *GPR143*/*OA1* carriers, pigmented and nonpigmented RPE cells segregate into radial arrays (29) not unlike the pattern of radially arranged dots observed in the UWF pseudocolor images (Fig. 3, *A* and *B*) of the current study. This similarity suggests that the pattern of dot-like lesions is established by RPE cells. Perhaps the reduction in melanin signal recorded by NIR-AF (27–30) reflects a remodeling of the RPE monolayer to fill gaps left by RPE loss. The involvement of RPE in the atrophy associated with *RLBP1*/CRALBP disease is indicated in other studies by the hyperfluorescence observed in fluorescein angiograms (9) and by the same hypertransmission of SD-OCT signal into the choroid at sites of atrophy (10). It has also been noted that the dot-like fundus lesions progress to RPE atrophy (6). Here the reduced melanin-derived NIR-AF signal was not restricted to the *RLBP1*/CRALBP-affected and Cralbp^−/−^ mice, instead this phenotype was also exhibited by the heterozygous carrier parents.

In the SD-OCT scans presented here the abnormal foci of hyperreflectivity in photoreceptor-attributable bands was limited to the IZ and EZ bands perhaps because of the younger ages of the affected siblings. In previous reports, however, these focal lesions incorporated EZ, external limiting membrane, and extended into the ONL (48) thus replacing photoreceptor cell-attributable bands. The degenerative changes in photoreceptor cells indicated by these SD-OCT findings are corroborated by the reduced ONL area in Cralbp^−/−^ mice.

The reduction in qAF in *RLBP1*/CRALBP-affected patients and Cralbp^−/−^ mice and in HPLC quantified bisretinoid in Cralbp^−/−^ mice is attributable to chronic deficiency in the 11-*cis*-retinal chromophore. Indeed we observed attenuated qAF even in the asymptomatic carrier parents carrying a single mutant allele. This finding indicates that a reduction of approximately half of the gene product reduces bisretinoid. Because bisretinoid lipofuscin accumulation underlies retinal diseases such as recessive Stargardt disease, various strategies aimed at reducing bisretinoid formation have been investigated in recent years (49–59). Here we show for the first time that slowing the visual cycle by a deficiency in CRALBP activity associated with biallelic mutations (patients) or even a mutation in one allele (parent carriers) also confers a decrease in bisretinoid lipofuscin. Moreover, our findings suggest that quantitation of autofluorescence may be used as a predictor of mutation severity as it showed a difference between the missense and deletion variants in the carriers.

Several findings in Cralbp^−/−^ mice replicated the disease features observed in the *RLBP1*/CRALBP-affected patients. Consistent with the abnormal ERG responses in the patients, 11-*cis*-retinal was significantly lower in Cralbp^−/−^ mice than in the WT. Measurement of SW-AF by qAF showed that fundus intensities were significantly lower in the Cralbp^−/−^ mice in keeping with the reduced qAF recorded in the patients. Moreover, HPLC quantitation of the bisretinoid visual cycle by-products, the source of SW-AF, was also reduced in the mutant mice. NIR-AF intensities originating primarily in RPE cells were found to be reduced in mice at 12 and 18 months of age and correspondingly, were reduced in the affected patients. Consistent with the central atrophy observed in RPA (10, 11) and with the SD-OCT evidence of structural aberration in photoreceptor cells observed here, we found that the numbers of photoreceptor cell nuclei spanning the width of ONL was decreased by 30% at age 12 months in Cralbp^−/−^ *versus* WT mice.

The CRALBP Phe96–99del mutation results in the loss of four residues, three of which are hydrophobic (Phe-96, Leu-97, and Phe-99) and one is charged (Arg-98); all are located on helix α4 of the N-terminal all-helical α domain. Although this region is ∼25 Å away from the ligand-binding pocket of CRALBP, insights into potential structure-function relationship can be gained from the R234W mutant crystal structure (23). The conformation of Arg-234 is essential for normal activity of CRALBP. Disruption of the Arg-234 conformation in the R234W mutant crystal structure resulted in a cascade of side chain conformational changes that ultimately affected the 11-*cis*-retinal–binding site and resulted in a 5-fold decrease in enzymatic activity.

**Figure 10.**
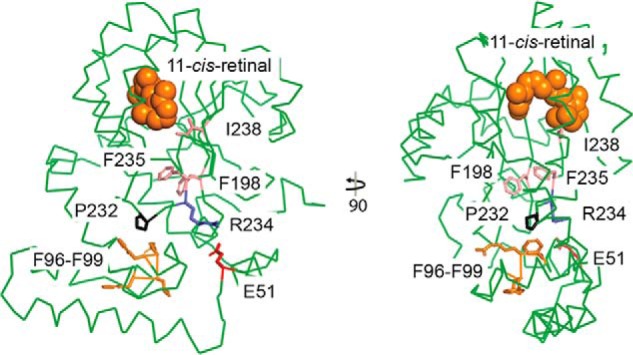
**Crystal structure of CRALBP.** Two views of the CRALBP crystal structure (PDB 3HY5) with key residues colored as indicated. Residues in *light pink* (I198, F235, and I238) underwent conformational change in the R234W mutant crystal structure (PDB 3HX3, not shown) to alter 11-*cis*-retinal binding affinity and decreased enzymatic activity. Residues lost in the Phe96–99del mutant are shown in *orange* (F96, L97, R98, and F99) and the interacting Pro-232 is shown in *black*. 11-*cis*-retinal is depicted by *orange spheres*.

The long-range effects of this single mutation at position Arg-234 (Fig. 10, *blue residue*) and the resulting structural rearrangements of three hydrophobic residues (Fig. 10, *light pink*) underscore the importance of this region for maintaining normal protein function. Deletion of residues 96–99 could alter the conformation of Arg-234 by preventing its interaction with Glu-51, with which it makes direct contact in the native crystal structure (C-O to *n* = O distance = 2.7 Å); this would also affect the retinal-binding pocket. Furthermore, Phe-99 interacts with Pro-232 (Fig. 10, *black*) and loss of Phe-99 could alter the conformation of the nearby Arg-234 by disruption of this interaction. These potential structural rearrangements within the Phe96–99del mutant provide a theoretical basis for decreased enzymatic activity through long-range disruption of the 11-*cis*-retinal–binding site, similar to that seen in the R234W crystal structure.

It is also possible that loss of the three hydrophobic residues Phe-96, Leu-97, and Phe-99, and the charged Arg-98 in the Phe96–99del mutation disrupt the structure and function through a different, unknown mechanism. For example, the absence of these residues could perturb nearby residues that make contacts critical for stabilizing the retinal-binding site or catalytic residues. Position 9 is in the disordered N terminus region of the crystal structure, so no structure-function relationship can be assessed for this region. Nevertheless, the R9C mutation could affect the pH dependence of enzymatic activity and this Arg-9 may be important in other conformational states.

What is the mechanism by which CRALBP deficiency causes RPE cell atrophy? The protein CRALBP in RPE cells serves as both an acceptor of 11-*cis*-retinol in the isomerization step of the visual cycle and as a facilitator of the oxidation of 11-*cis*-retinol to 11-*cis*-retinaldehyde (16, 60, 61). By chaperoning 11-*cis*-retinal it also protects against damage from free aldehyde. Whether the loss of the retinaldehyde-carrier function of CRALBP in the presence of regenerated 11-*cis*-retinal is permissive for unwanted aldehyde toxicity, remains to be determined in our ongoing studies.

The current qAF findings are of additional interest. It was noted previously that Cralbp^−/−^ mice are protected from light damage (1) and it was suggested that Cralbp deficiency reduces the quantity of the mediator of light-mediated retinopathy (1). Based on age and genotype associations we have previously presented evidence implicating bisretinoids as the initiators of light damage (62). The reductions in bisretinoid and retinoid reported here likely reveal the mechanism by which *RLBP1*/CRALBP deficiency protects against light damage.

For the most part, treatments for arRP are not available. However, gene therapy currently exists for patients carrying *RPE65* mutations (63–65) that share some clinical features with *RLBP1*/CRALBP disease. Moreover, subretinal injection of an adeno-associated vector driving the expression of human CRALBP in Cralbp^−/−^ mice improved both cone and rod electroretinographic responses (66, 67).

In summary, with deficiency in CRALBP, the cone and rod visual cycles fail to provide normal levels of visual chromophore. The latter deficiency manifests in *RLBP1/*CRALBP-affected patients as nonrecordable rod ERG responses that recover after prolonged dark adaptation. The chronically diminished 11-*cis*-retinal is realized as pronounced declines in bisretinoid visual cycle by-products, the source of SW-AF, in *RLBP1/*CRALBP-affected patients, and with lesser declines in symptomless *RLBP1/*CRALBP carriers. The reductions in qAF and NIR-AF intensities in the affected patients and in their heterozygous parents have not previously been reported. Indeed these decreases in qAF and NIR-AF are indicative of RPE changes in heterozygous carriers and signify a phenotype not previously recognized in humans heterozygous for *RLBP1/*CRALBP mutations.

## Experimental procedures

### Human patients

The patients presented to the Department of Ophthalmology, Columbia University Medical Center. Each patient had a complete ophthalmic examination that included best-corrected visual acuity, tonometry and anterior segment, media and fundus examination. Pupils were dilated with topical 1% tropicamide and 2.5% phenylephrine before imaging and fundus examination. The patients provided written informed consent under a protocol approved by the Institutional Review Board of Columbia University. The study was approved by the Institutional Review Board at Columbia University and adhered to tenets established by the Declaration of Helsinki.

### Electroretinography

ffERG were performed in the patients according to the standards from the International Society for Clinical Electrophysiology (ISCEV) using the Diagnosys Espion Electrophysiology system (Diagnosys LLC, Lowell, MA) and Dawson Trick Litzkow (DTL) fiber electrodes in both scotopic and photopic states.

### Fundus imaging

Horizontal SD-OCT scans (9 × 9 mm scans; 870 nm; 7 μm axial resolution) through the macula were acquired by cSLO (confocal scanning laser ophthalmoscopy; Spectralis HRA+OCT, Heidelberg Engineering, Heidelberg, Germany) in high-resolution mode with an average of 100 single scans. The scans were registered automatically to a simultaneously acquired IR reflectance (IR-R) (820 nm) fundus image. Nomenclatures used to identify reflectivity bands in SD-OCT images were as published (68).

SW-AF images (488 nm excitation; emission 500 to 680 nm; 30° × 30° field) were obtained using a cSLO (Spectralis) HRA+OCT (Heidelberg Engineering, Heidelberg, Germany). For qAF, images were acquired and analyzed as described previously (69–72) using a Spectralis HRA+OCT equipped with an internal fluorescent reference for correction of variable laser power and differences in detector sensitivity. The images were acquired in high-speed mode (8.9 frames/s), as a minimum of 12 frames (video format) and saved in nonnormalized mode. A minimum of 6 frames per video was required for the analysis. Two sessions of three videos each were performed per eye. An averaged nonnormalized image was generated from each video and the two best images from each session were analyzed (4 averaged images per eye).

Mean gray levels were determined in eight circular segments, positioned 7 to 9° from the fovea. Vessels in the sampling area, which would decrease the qAF level, were accounted for by the software algorithm. qAF values were calculated after gray levels were calibrated to gray levels in the reference; and after accounting for the zero-gray level of the laser, refractive error, image magnification, and age-adjusted lens transmission (73). For each eye, a qAF value was computed as the mean of the qAF values of the segments (qAF_8_). The healthy control group (74) was composed of 87 subjects of white ethnic background with an age range of 5 to 58 years old. Color-coded qAF maps were computed based on pixel-wise transformation of qAF values (WaveMetrics, Lake Oswego, OR).

NIR-AF (790 nm excitation, >830 nm emission, 30 × 30° field) images were obtained with a Heidelberg Retina Angiograph 2 scanning laser ophthalmoscope (HRA2-cSLO, Heidelberg Engineering, Heidelberg, Germany) using the indocyanine-green angiography mode. To obtain high-quality images, the eye-tracking function was utilized, and 100 single frames were averaged and saved in normalized mode. Semi-quantitative analysis of NIR-AF imaging was also performed. With a sensitivity of 96 (within a range of 51–100%) and the eye-tracking function, 100 single frames were averaged to obtain high-quality images saved in nonnormalized mode. ImageJ (RRID:SRC_003070; provided in the public domain by the National Institutes of Health, Bethesda, MD) was used to analyze and plot the NIR-AF signal. Nineteen subjects (mean age 35.96 years) without a history of eye disease served as the healthy-eye group. These individuals self-identified as Caucasian (11), African American (3), Asian (3), and Hispanic (2).

UWF pseudocolor images were acquired using the Optos 200 Tx imaging system (Optomap Daytona, United Kingdom, Scotland; lasers: red, 633 nm; and green, 532 nm) with red and green false-color display. UWF-AF/green were also acquired (532 nm excitation; 570–780 emission; 200° field).

For analysis, all images were registered and aligned using i2k Retina software (Dual Align LLC, Clifton Park, NY). For illustrative purposes, some nonaligned images were used in figures.

### Mice

Cralbp^−/−^ mice (Rpe65–Leu-450; agouti) were obtained as a gift from Dr. Vladimir J. Kefalov, and were bred in-house. 129S1/SvImJ mice (Rpe65–Leu-450; agouti) and Rpe65^−/−^ mice (Rpe65^Rd12^; black) were purchased from The Jackson Laboratories, Bar Harbor, ME. Animal protocols were approved by the Institutional Animal Care and Use Committee of Columbia University and complied with guidelines set forth by the ARVO Animal Statement for the Use of Animals in Ophthalmic and Vision Research. Mice were free of the Crb1/Rd8 mutation.

### Analysis of retinoid in mice eyes

For UPLC quantitation of retinoids, frozen mouse eyecups (1 eye/sample) were homogenized and derivatized with *O*-ethylhydroxylamine (100 mm; 1 ml, on ice) in Dulbecco's phosphate-buffered saline (pH 6.5, without CaCl_2_ and MgCl_2_) using glass homogenizers. After vortexing, samples were allowed to stand for 15 min at 4 °C. All-*trans*-retinyl acetate was added to each sample as an internal standard. Methanol (1 ml) was added and the samples were vortexed and extracted with hexane (3 ml, 3 times). After centrifugation (1500 × *g* for 5 min at 4 °C), the hexane phase was dried under argon gas and then resuspended in 20 μl of acetonitrile. For separation, a Waters Acquity UPLC-PDA system (Waters, Milford, MA) was used with a CSH C18 column (1.7 μm, 2.1 × 100 mm) and gradients of water (A) and acetonitrile (B) with 0.1% of formic acid as follows: starting at 60% B; holding for 5 min; a linear increase to 70% B over 55 min; a linear increase to 100% B over 10 min; holding for 20 min. The flow rate was 0.3∼0.5 ml/min. RAL (*O*-ethyl) oximes were monitored at 360 nm and all-*trans*-retinol and all-*trans*-retinyl palmitate were monitored at 320 nm. UV absorbance peaks were identified by comparison with external standards of synthesized 11-*cis*-retinal (*O*-ethyl) oxime, all-*trans*-retinal (*O*-ethyl) oxime, all-*trans*-retinol, and all-*trans*-retinyl palmitate. Molar quantities per eye were calculated based on standard solutions with concentrations determined spectrophotometrically. Peak areas were calculated using Waters Empower Software and results were analyzed in Excel (Microsoft, Redmond WA).

### qAF in mice

Mice were anesthetized, pupils were dilated, the cornea was lubricated, and mice were positioned as previously described (75–77). SW-AF images (488 nm, 55° wide-field lens; 0.98 mm detection pupil) were captured with a cSLO (Spectralis HRA; Heidelberg Engineering, Heidelberg, Germany) with laser power set at ∼280 microwatts and sensitivity at 100–105, after visual pigment was bleached for 20 s. Nine successive frames were acquired in SW-AF high-speed mode and were saved as nonnormalized images. Mean gray levels (GL) in the SW-AF images were measured in 8 predefined segments and qAF at 488 nm excitation was calculated by normalization to the GL of the reference after subtraction of zero light (GL_0_) and inclusion of a reference calibration factor.

A mean of 100 frames were also obtained at 790 nm excitation (NIR-AF) with high resolution ART mode (Automatic Real-Time) and resized with Photoshop CS4 (Adobe Systems, San Jose, CA) to 768 × 768 pixels (equivalent to high-speed mode). Intensity at 790 nm was calculated by subtracting the minimal GL of optic nerve head measured by ImageJ (a public domain, Java-based image processing program developed at the National Institutes of Health).

### Measurement ONL

Mouse eyes were marked superiorly with tattoo ink (Ketchum Manufacturing Inc., Ottawa, Canada), fixed in alcoholic Z-fix (4% paraformaldehyde, 20% isopropyl alcohol, 2% TCA, and 2% zinc chloride), and paraffin sections (5 μm) were stained with H&E. Using digitized images of three sections traversing the optic nerve head (ONH), nuclei in rows spanning the width of the ONL were counted at 200-μm intervals superior and inferior to the ONH along the vertical meridian (0.2 to 1.6 or 2 mm) and the ONL area was calculated using a measurement interval of 0.2 mm multiplied by the sum of ONL thicknesses in superior and inferior hemiretina (ONH to 2.0 mm).

### Bisretinoid analysis in mice

Mouse eyecups (4–10 eyes/sample) were homogenized, extracted in chloroform/methanol, and concentrated as previously described (78, 79). Then the indicated mouse eyes were freeze-dried before extraction. For UPLC quantitation, murine eyecup samples were re-dissolved in ethanol after extraction and injected (10 μl) into a Waters Acquity UPLC-MS system (Waters, Milford, MA) using an Acquity BEH Phenyl Column (1.7 μm, 2.1 × 100 mm; Waters, Milford, MA). Eluents were water/acetonitrile (1:1) with 0.1% formic acid (A) and isopropyl alcohol/acetonitrile (9:1) with 0.1% formic acid (B). Separation was achieved using the following elution gradients: 0–50 min, 100–55%; 50–110 min, 55–35%. Flow rate was 0.2 ml/min. UV absorbance peaks were identified by comparison with external standards of synthesized and purified A2E and A2GPE. Identification was confirmed by mass to charge ratio (*m*/*z*). Molar quantities per eye were calculated based on standard solutions with concentrations determined spectrophotometrically. Peak areas were calculated using Waters Empower Software and results were analyzed in Excel (Microsoft, Redmond WA).

For analysis by HPLC (Alliance System, Waters Corp.), samples were re-dissolved in CHCl_3_/MeOH = 1:1 after extraction as above and injected (30 μl) as previously described (80). Absorbance peaks were identified using synthesized authentic compounds, and molar quantities per eye were calculated by comparison with synthesized standards. A2E and iso-A2E were measured separately and summed.

### Structural analysis

Structural figures were made in PyMOL using CRALBP crystal structure (PDB 3HY5) (81).

### Statistical analysis

*p* values were calculated using PRISM 8 (GraphPad Software).

### Data availability

All of the data are in the manuscript.

## Author contributions

J. R. L. d. C., H. J. K., K. U., J. Z., A. P. O., and T. Y. investigation; J. R. L. d. C., H. J. K., K. U., J. Z., and J. R. S. writing-original draft; H. J. K., K. U., and J. Z. formal analysis; S. H. T. and J. R. S. funding acquisition; S. H. T. and J. R. S. project administration; J. R. S. conceptualization; J. R. S. writing-review and editing.

## Footnotes

3 The abbreviations used are:

CRALBP: cellular retinaldehyde-binding protein
cSLO: confocal laser scanning ophthalmoscopy
ffERG: full-field electroretinography
NIR-AF: near-infrared fundus autofluorescence
ONL: outer nuclear layer
RDH5: retinol dehydrogenase 5
RLBP1: retinaldehyde-binding protein 1
RPA: retinitis punctata albescens
RPE: retinal pigment epithelium
SD-OCT: spectral domain-optical coherence tomography
SW-AF: short-wavelength fundus autofluorescence
UWF: ultrawide-field
qAF: quantitative fundus autofluorescence
UPLC: ultra-performance LC
ANOVA: analysis of variance
ONH: optic nerve head
Rt: retention time.
